# Low-Latency Spiking Neural Networks Using Pre-Charged Membrane Potential and Delayed Evaluation

**DOI:** 10.3389/fnins.2021.629000

**Published:** 2021-02-18

**Authors:** Sungmin Hwang, Jeesoo Chang, Min-Hye Oh, Kyung Kyu Min, Taejin Jang, Kyungchul Park, Junsu Yu, Jong-Ho Lee, Byung-Gook Park

**Affiliations:** Inter-university Semiconductor Research Center (ISRC) and Department of Electrical and Computer Engineering, Seoul National University, Seoul, South Korea

**Keywords:** spiking neural networks, low-latency, fast inference, pre-charged membrane potential, delayed evaluation

## Abstract

Spiking neural networks (SNNs) have attracted many researchers’ interests due to its biological plausibility and event-driven characteristic. In particular, recently, many studies on high-performance SNNs comparable to the conventional analog-valued neural networks (ANNs) have been reported by converting weights trained from ANNs into SNNs. However, unlike ANNs, SNNs have an inherent latency that is required to reach the best performance because of differences in operations of neuron. In SNNs, not only spatial integration but also temporal integration exists, and the information is encoded by spike trains rather than values in ANNs. Therefore, it takes time to achieve a steady-state of the performance in SNNs. The latency is worse in deep networks and required to be reduced for the practical applications. In this work, we propose a pre-charged membrane potential (*PCMP*) for the latency reduction in SNN. A variety of neural network applications (e.g., classification, autoencoder using MNIST and CIFAR-10 datasets) are trained and converted to SNNs to demonstrate the effect of the proposed approach. The latency of SNNs is successfully reduced without accuracy loss. In addition, we propose a delayed evaluation method (*DE*), by which the errors during the initial transient are discarded. The error spikes occurring in the initial transient is removed by *DE*, resulting in the further latency reduction. *DE* can be used in combination with *PCMP* for further latency reduction. Finally, we also show the advantages of the proposed methods in improving the number of spikes required to reach a steady-state of the performance in SNNs for energy-efficient computing.

## Introduction

In recent years, analog-valued neural network (ANN) has achieved the great success in various fields such as image recognition, natural language processing, autonomous vehicle, etc. (LeCun et al., 2015; Schmidhuber, 2015; Li et al., 2015; Chen et al., 2015; Krizhevsky et al., 2017). Nevertheless, due to the enormous power consumption required for inference, interests in new types of neural networks have been developed (Han et al., 2015; Hubara et al., 2016; Yang et al., 2017; Cao et al., 2015; Hubara et al., 2017). Spiking neural network (SNN) based on more biologically plausible neuron models has been considered as the third generation of artificial neural network (Maass, 1997; Ghosh-Dastidar and Adeli, 2009; Paugam-Moisy and Bohte, 2012). Since SNN is an event-driven system where a neuron updates its state only when there is a spike, it is promising especially when implementing in neuromorphic hardware due to its high energy-efficiency (Indiveri et al., 2015; Merolla et al., 2014; Seo et al., 2011); however, SNN has not been widely used because of the lack of learning algorithms that can achieve high performance comparable to ANN (O’Connor and Welling, 2016; Tavanaei et al., 2018; Ponulak and Kasiński, 2011; Iakymchuk et al., 2015). Recently, many studies on spiking neural networks (SNNs) have achieved almost the same performance as ANNs by mapping the trained weight from ANNs to SNNs (Diehl et al., 2015; Rueckauer et al., 2017). In evaluating the performance of SNNs, not only accuracy but also latency is of great importance (Diehl et al., 2015; Rueckauer and Liu, 2018; Hu et al., 2018; Sengupta et al., 2019). Unlike ANN where outputs are obtained as soon as inputs are applied, there is the latency for SNN to achieve the best performance because a signal is transmitted only when a spike is generated (Webb and Scutt, 2000; Diehl et al., 2015; Stromatias et al., 2015; [Bibr B31][Bibr B1]). That is, in order for a neuron in a certain layer to fire, synaptic integration and spike generation must be carried out sequentially in all the preceding layers, resulting in the latency. One more thing causing the latency is that information is encoded by rate-based coding in most SNN models. Thus, it takes time for spikes to represent the equivalent precision to ANN’s activations. The latency of SNN models where information is encoded by other than rate-based coding such as temporal coding, phase coding, and etc., may be short compared with that of models using rate-based coding (Gütig and Sompolinsky, 2006; Ponulak and Kasiński, 2010; Zhang et al., 2018; Zenke and Ganguli, 2018). However, the latency is inevitable in SNN regardless of the spike encoding method, and it can be worse as the scale of network increases. Performing inference operations over sufficiently long time will result in high accuracy, but it entails large power consumption and slow inference speed. In order to utilize SNNs in practical applications, therefore, it is important to achieve high accuracy within a short time. Neil et al. (2016) have reported algorithms for low-latency SNNs using on rate-based coding, but it was an applicable method at training-level. For SNNs using rate-based coding, latency reduction method at inference-level has not been reported.

In this paper, we propose a pre-charged membrane potential (*PCMP*) for fast inference in SNN by which a membrane potential is charged to a certain level prior to the beginning of the inference operation. *PCMP* reduces an SNN error at the early timesteps by inducing the earlier firing of the first spike, so it can improve the latency until the best performance is achieved. We also introduce a technique to achieve additional latency reduction by discarding some spikes occurring at the initial timesteps, referred as a delayed evaluation (*DE*). Spikes that occur at the initial timesteps are likely to contain inaccurate information; therefore, paradoxically, adding a deliberate delay in the inference operation results in the latency reduction. The feasibility of the proposed methods is verified by model equations of SNNs. Then, we demonstrate the effect of the proposed methods using classifiers and autoencoder for image compression and decompression. Moreover, the reduction of the latency can lead to a less synaptic operation required to reach the steady-state of the performance. We show the energy efficiency of the proposed methods in the neural network applications. All the SNN simulations in this work are conducted by *PyTorch* (*ver. 1.0.0*).

## Materials and Methods

### Overview of the Proposed Methods

In the conventional analog-valued neural network (ANN), a neuron corresponding to an activation function performs summation of signals multiplied by weights from synapses connected in parallel, which denotes spatial integration (Rosenblatt, 1958). Since the concept of time is excluded in ANN, the output is obtained as soon as the input is applied. On the contrary, there is the latency to achieve the best performance in SNN caused by the following two reasons: (1) neuron carries out temporal integration as well as spatial integration, so the synaptic integration and spike generation must be carried out sequentially in all preceding layers to get the output spikes, and (2) unlike ANN where the value of activation represents information, the value is represented by the number of spikes per given timespan in SNN models using rate-based coding, so that it takes time to obtain a precision comparable to that of the ANN activation (Cardarilli et al., 2013; Rullen and Thorpe, 2001; Thorpe et al., 2001).

The latency is an inherent issue of SNNs, so it can occur regardless of the implementation method; In order to demonstrate the effectiveness of this work, however, we utilize an ANN-to-SNN conversion method since it is one of the most effective way to implement high-performance SNNs. The ANN-to-SNN conversion method is based on one-to-one correspondence between an ANN neuron and a spiking neuron, so a ReLU activation can approximate a firing rate of a spiking neuron (Rueckauer et al., 2017; Hu et al., 2018). The ReLU activation of ANN for *i*-th neuron in layer *l* is defined as,

(1) $$a_{i}^{l}=\max\left(0,\sum_{j=1}^{M^{l-1}}w_{i\,j}^{l}\,a_{j}^{l-1}+b_{i}^{l}\right),$$

where *M^l^* is the number of neuron in layer *l*, $w_{i\,j}^{l}$ is weight from *j*-th neuron in layer *l-1* to *i*-th neuron in layer *l*, and $b_{i}^{l}$ is bias. For layer *l=0*, $a_{i}^{0}$ corresponds to the input. Based on the model equations of SNN using rate-based coding in the previous studies, the firing rate $r_{i}^{l}$ of the *i*-th neuron in layer *l* can be expressed as a function of time *t* and the initial membrane potential $V_{i}^{l}\,\left(0\right)$(Hwang et al., 2020).

(2) $$r_{i}^{l}\left(t,V_{i}^{l}\left(0\right)\right)=\frac{N_{i}^{l}\,\left(t,V_{i}^{l}\,\left(0\right)\right)}{t}=\frac{1}{V_{\text{th}}}[\sum_{j=1}^{M^{l-1}}w_{i\,j}^{l}r_{j}^{l-1}\left(t,V_{i}^{l}\left(0\right)\right)+r_{\max}b_{i}^{l}-\frac{V_{i}^{l}\,\left(t\right)-V_{i}^{l}\,\left(0\right)}{t}].$$

The definitions of symbols are summarized in Table 1. Setting *V*_*th*_ to 1 for simplicity, the firing rate of a spiking neuron in the input layer (*l=0*) is related with the ANN inputs $a_{i}^{0}$, which can be expressed as,

**TABLE 1:** Definition of symbols.

**Symbol**	**Definition**
*t*	Time
*l*	Layer index
*i*	Neuron index
*$w_{i\,j}^{l}$*	Weight
*$b_{i}^{l}$*	Bias
$a_{i}^{l}$	ReLU activation
*$V_{i}^{l}\,\left(t\right)$*	Membrane potential
$r_{i}^{l}\,\left(t,V_{i}^{l}\,\left(0\right)\right)$	Firing rate
$N_{i}^{l}\,\left(t,V_{i}^{l}\,\left(0\right)\right)$	The total number of spikes during *t*
*r*_*max*_	Maximum firing rate
*M^l^*	Number of neurons in layer *l*
*V*_*th*_	Threshold of a neuron
$\varepsilon_{i}^{l}$	Partial SNN error
$E_{i}^{l}\,\left(t,V_{i}^{l}\,\left(0\right)\right)$	SNN error
*V*_*pc*_	Pre-charged membrane potential

(3) $$r_{i}^{0}\,\left(t,V_{i}^{0}\,\left(0\right)\right)=a_{i}^{0}-\frac{V_{i}^{0}\,\left(t\right)-V_{i}^{0}\,\left(0\right)}{t}.$$

For consistency, we assume that the input spikes are generated by the same method as in the other neural network layers. Starting with Eq. (3), we can recursively expand Eq. (2) as,

$$r_{i}^{l}\,\left(t,V_{i}^{l}\,\left(0\right)\right)=a_{i}^{l}+\varepsilon_{i}^{l}+\sum_{i_{l-1}=1}^{M^{l-1}}w_{i_{l}\,i_{l-1}}^{l}\,\varepsilon_{i_{l-1}}^{l-1}+\cdots +\sum_{i_{l-1}}^{M^{l-1}}w_{i_{l}\,i_{l-1}}^{l}\,\cdots$$

(4) $$\sum_{i_{0}=1}^{M^{0}}w_{i_{1}\,i_{0}}^{1}\,\varepsilon_{i_{0}}^{0}.$$

Here, if $\sum_{j=1}^{M^{l-1}}w_{i\,j}^{l}\,r_{j}^{l-1}\,\left(t,V_{i}^{l}\,\left(0\right)\right)+r_{\max}\,b_{i}^{l}>0$, a partial error $\varepsilon_{i}^{l}$ is $-\frac{V_{i}^{l}\,\left(t\right)-V_{i}^{l}\,\left(0\right)}{t}$; otherwise, it is zero since the neuron does not generate any spike. From Eq. (4), we can define an SNN error as,

$$E_{i}^{l}\,\left(t,V_{i}^{l}\,\left(0\right)\right)=r_{i}^{l}\,\left(t,V_{i}^{l}\,\left(0\right)\right)-a_{i}^{l}=\varepsilon_{i}^{l}+\sum_{i_{l-1}=1}^{M^{l-1}}w_{i_{l}\,i_{l-1}}^{l}\,\varepsilon_{i_{l-1}}^{l-1}+\cdots +$$

(5) $$\sum_{i_{l-1}=1}^{M^{l-1}}w_{i_{l}\,i_{l-1}}^{l}\,\cdots \,\sum_{i_{0}=1}^{M^{0}}w_{i_{1}\,i_{0}}^{1}\,\varepsilon_{i_{0}}^{0}.$$

Eq. (5) says that the SNN error of layer *l* is the sum of the partial error generated in that layer and the partial errors generated at and propagated through the previous layers.

Up to the present, it has been customary to set all initial membrane potentials, $V_{i}^{l}\,\left(0\right)$, to zero before the inference starts. If $V_{i}^{l}\,\left(0\right)=0$, however, the partial error, $\varepsilon_{i}^{l}$, is always negative, resulting in the inherent latency of SNN. If we set $V_{i}^{l}\,\left(0\right)$ to some positive value, *V*_*pc*_ (< *V*_th_), it corresponds to *PCMP*. Since $\frac{V_{\text{pc}}}{t}$ will compensate the negative value of the term, $\frac{V_{i}^{l}\,\left(t\right)}{t}$, it can have a strong impact on the SNN error during the initial transient.

Some studies have reported that the method of lowering *V*_*th*_ to reduce the latency by increasing the firing rate (Diehl et al., 2015; Park et al., 2019). As shown in Eq. (2), the decrease of *V*_*th*_ without considering the threshold balancing leads to the increase of the steady-state firing rate, resulting in a large error propagating to the subsequent layers. Thus, the degradation in the steady-state of performance can occur. However, from Eq. (4), we can notice that *PCMP* does not alter the steady-state firing rate because $\varepsilon_{i}^{l}$ decays to zero as the inference time increases. In other words, *PCMP* only induces an earlier firing of the first spike. If the integration starts from *V*_*pc*_, the potential required to reach the threshold of a neuron (*V*_*th*_) is reduced to *V*_*th*_ −*V*_pc_, resulting in earlier firing of the first spike. After the first spike, the membrane potential is reset by subtraction so that the neuron starts to generate spikes with almost regular time interval (Rueckauer et al., 2017). Therefore, *PCMP* is powerful in that it only can reduce the latency without violating the equivalence condition between the ANN activations and the firing rates of SNN.

On the other hands, the firing rate $r_{i}^{l}\,\left(t,V_{i}^{l}\,\left(0\right)\right)$ generates low-precision information and contains a considerable error at the early timesteps based on Eq. (4). As mentioned in Eq. (5), the error propagates to the subsequent layers multiplied by the weights, so it becomes significantly large in the output layer. Of course, the first few spikes play a significant role in biological nerve systems, and other spike coding methods such as temporal or first-time-to-spike coding are actively studied (Rullen and Thorpe, 2001; Park et al., 2020); however, it is likely that the initial few spikes occurring in the output neurons entail inaccurate information when rate-based coding is used. Thus, we try to discard the error spikes at the early timesteps by giving deliberate delay in the output neurons, referred as a *DE*. This method can be utilized in combination with *PCMP* to achieve further latency reduction.

### Network Architecture

Firstly, we train a network composed of 3**C**20-5**C**50-**FC**50-**FC**10 for MNIST classification, referred as *Net 1*. *n***C***m*(*s*) represents *n* × *n* convolution operation with *m* filters and stride *s*, and **FC***n* means a fully connected layer with *n* neurons. Dropout technique is employed with the probability of 50%, and the adaptive learning rate by multiplying 0.1 after 60, 120, and 180 epochs with the initial value of 1 × 10^–3^ is applied. Weights are optimized by Adam with L2 decay parameter of 1 × 10^–4^. After training, a test accuracy of 99.35% is achieved.

*Net 2* is an all convolutional network consisting of 3**C**96-3**C**96(2)-3**C**192-3**C**192-3**C**192(2)-3**C**192-1**C**192-1**C**10-**GAP** where **GAP** denotes for global average pooling (Springenberg et al., 2014). Some nodes are randomly dropped out with the probability of 50% for generalization, and the adaptive learning rate by multiplying 0.5 after 50, 100, and 200 epochs with the initial value of 1 × 10^–3^ is applied. Stochastic gradient descent (SGD) algorithm is utilized for optimization. The classification accuracy of 91.77% is obtained for test dataset.

In order to demonstrate the effect of the proposed methods on general SNN applications, *Net 3* is trained as an autoencoder. The structure is 3**C**96-3**C**96(2)-3**C**32(2)-3**C**16(2)-3**C^*T*^**32(2)-3**C^*T*^**96(2)-3**C^*T*^**96(2)-3**C^*T*^**3 where *n***C^*T*^***m*(*s*) represents *m* transposed convolution filters of size *n* × *n*. CIFAR-10 dataset is employ to train the network with Adam optimizer and the adaptive learning rate by multiplying 0.1 after 120, 240, and 360 epochs with the initial value of 1 × 10^–3^. Lena image (512 × 512) split into 256 of 32 × 32 patches is used as a test sample for inference. Detailed training method and hyper parameters are based on the previous work (Hwang et al., 2020).

The trained weights are normalized by “Data-based Normalization” and transferred to SNNs (Diehl et al., 2015). We employ the rate-based coding to represent ANN input activations by applying constant current inputs to the integrate-and-fire neurons. The simulation timestep is divided to simulate the SNNs most efficiently in the aspect of the computing complexity so that a neuron with the highest firing rate generates spikes in every timestep. The ANN-to-SNN conversion used in this work is based on the previous work (Rueckauer et al., 2017).

## Results

### Latency of SNNs

**FIGURE 1:**
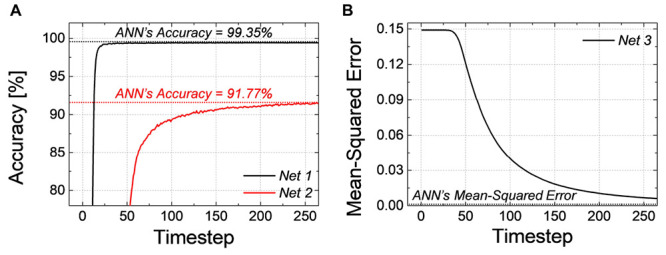
**(A)** Accuracy of *Net 1*, *2* and **(B)** MSE of *Net 3* according to the simulation timestep. Time to reach ANN’s performance is required because of the latency of SNN.

The classification accuracy and *MSE* of SNNs according to the simulation timestep for *Net 1* ∼ *3* are shown in Figures 1A,B, respectively. As the simulation time increases, the performance of SNNs increases as well, and it converges to the ANN’s performance. As shown in Figure 1A, for *Net 1*, which represents a relatively simple network, it reaches ANN’s accuracy within a small number of timesteps; however, in a much deeper network, such as *Net 2*, the latency becomes severely worse resulting in a longer convergence time. We also extract *MSE* of *Net 3* between the SNN firing rate and ANN’s activation in the output layer, so zero *MSE* means that the SNN’s output equals to the ANN’s output. As illustrated in Figure 1B, *MSE* of *Net 3* gradually decreases with respect to the number of timesteps, but *MSE* does not reach ANN’s performance within 255th timestep.

**FIGURE 2:**
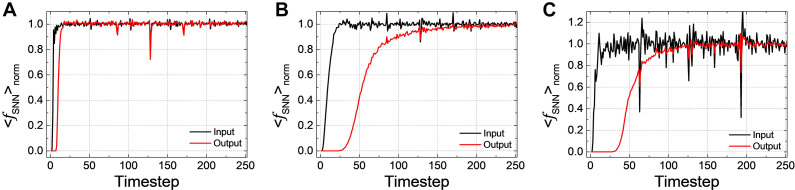
< *f*_*SNN*_ > *_norm_*, the averaged firing rate over all the test samples normalized by ANN’s activation in the input and output layers according to the simulation timestep for **(A)** *Net 1*, **(B)** *Net 2*, and **(C)** *Net 3*. < *f*_*SNN*_ > *_norm_* of the output layer suffers from the latency compared with that of the input layer.

The latency of SNN can be observed in < *f*_*SNN*_ > _*norm*_, the averaged firing rate over all test samples normalized by ANN’s activation. < *f*_*SNN*_ > _*norm*_ of *Net 1* ∼ *3* in the input and output layers according to the simulation timestep are shown in Figures 2A–C, respectively. If < *f*_*SNN*_ > *_norm_* converges to one, it indicates that the performance of SNNs becomes equivalent to that of ANNs. We can see that even < *f*_*SNN*_ > *_norm_* of the input layer has some latency until it converges to one (it is quite small in *Net 1*, but very conspicuous in *Net 2* and *3*). < *f*_*SNN*_ > *_norm_* of the output layer is lagging behind that of the input layer due to the latency, and it is worse in the deep and complex network like *Net 2* and *3*.

### Pre-charged Membrane Potential

**FIGURE 3:**
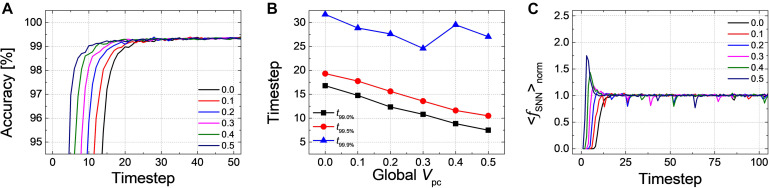
**(A)** Result of accuracy-timestep for *Net 1* with the global *V*_*pc*_ varying from 0.0 to 0.5 with 0.1 steps. **(B)** Extracted timesteps (*t*_99.0%_, *t*_99.0%_, and *t*_99.9%_) with respect to the amount of the global *V*_*pc*_. **(C)** The average firing rate in the output layer with the different levels of the global *V*_*pc*_ according to the simulation timestep.

Firstly, we have simulated the effect of *PCMP* by applying the global *PCMP* (the same initial membrane potential, *V*_*pc*_, to all neurons in the network). Figure 3A illustrates the classification accuracy as a function of simulation time for *Net 1*, while varying *V*_*pc*_ from 0.0 to 0.5 with 0.1 steps. As *V*_*pc*_ increases, there seems fast convergence to the ANN’s accuracy in that the accuracy vs. time curve shifts to the left. In order to precisely estimate the latency reduction, the number of timesteps to reach 99.0, 99.5, and 99.9% of the ANN’s accuracy (denoted as *t*_99.0_, *t*_99.5_, and *t*_99.9%_) are extracted, as shown in Figure 3B. The timestep to reach 99.0 and 99.5% of the ANN’s accuracy is decreased with the increase of *V*_*pc*_ from 0.0 to 0.5. If the criterion of latency is raised to 99.9% of the ANN’s accuracy, *PCMP* up to 0.3 reduces the latency while too much *PCMP* increases it. *PCMP* can substantially improve the convergence time so that we can obtain the best latency reduced by 59% for *t*_99.0%_ (@*V*_*pc*_ of 0.5), 43% for *t*_99.5%_ (@*V*_*pc*_ of 0.5) and 25% for *t*_99.9%_ (@*V*_*pc*_ of 0.3) compared with the case without *PCMP* (@*V*_*pc*_ of 0.0). Figure 3C shows < *f*_*SNN*_ > *_norm_* of *Net 1* in the output layer with respect to the different levels of *V*_*pc*_. The point of the rapid rise in < *f*_*SNN*_ > _*norm*_ moves to the left as *V*_*pc*_ increases, but too much *V*_*pc*_ induces over-firing during the early timesteps, which can rather degrade the latency.

**FIGURE 4:**
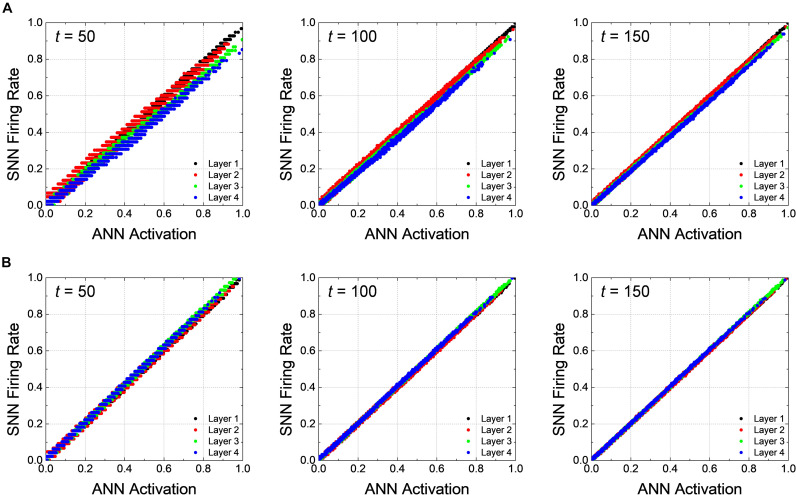
Correlation plots of *Net 1* at 50, 100, and 150th timesteps for the global *V*_*pc*_ of **(A)** 0.0 and **(B)** 0.5. The latency occurs in early timestep, and its effect gradually disappears along with the simulation time. Latency reduction effect is more noticeable when the global *V*_*pc*_ of 0.5 is applied.

In addition, correlation diagrams which reveal that *V*_*pc*_ facilitates the rapid inference in SNNs by reducing inherent delay are extracted in Figure 4. How precisely the firing rates of SNNs reproduce the ANN activations can be analyzed through the correlation diagram, where an ANN activation of a neuron is on *x*-axis while its SNN firing rate is on *y*-axis (Rueckauer et al., 2017; Hwang et al., 2020). If the SNN firing rate is perfectly reproduce the ANN activation for all neurons, all points in the correlation diagram are on line (*y* = *x*); however, when there is the latency, the firing rate of SNNs falls behind the activation of ANNs; thus, the points on the correlation diagram are positioned below the line of perfect correlation (*y* = *x*). The correlation diagrams are extracted from all neurons in the network using test dataset at 50, 100, and 150th timesteps with *V*_*pc*_ of 0.0 and 0.5, as shown in Figures 4A,B, respectively. In both cases, the firing rates of the neurons close to the input layer correlate quite well with the ANN activations even at the early time. As the simulation time increases, the firing rates of the neurons close to the output layer converges to the ANN activations, so that the points tend to be on the line (*y* = *x*); however, the increase of the slope is much faster for *V*_*pc*_ of 0.5. The slope of the correlation diagram of the output layer for *V*_*pc*_ of 0.0 is still less than one at the 150th timestep, showing that there still remains some delay.

**FIGURE 5:**
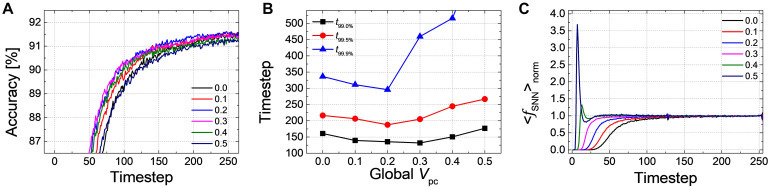
**(A)** Accuracy-timestep result for *Net 2* with the global *V*_*pc*_ changing from 0.0 to 0.5 with 0.1 steps. **(B)** Extracted timestep to reach 99.0, 99.5, and 99.9% of the ANN’s accuracy according to the changes of the global *V*_*pc*_. **(C)** The average firing rate in the output layer with different level of the global *V*_*pc*_ according to the simulation timestep. It is noticeable that the point of timestep in which the firing rate rises becomes earlier.

For *Net 2*, Figure 5A shows the changes of the classification accuracy according to the inference time with the different levels of *V*_*pc*_. The time-accuracy curve prominently shifts to the left with the increase of *V*_*pc*_ from 0.0 to 0.3 while it goes back to the right when *V*_*pc*_ above 0.3 is applied. The timestep when the accuracy reaches to 99.0, 99.5, and 99.9% of the ANN’s accuracy is extracted with respect to the changes of *V*_*pc*_ in Figure 5B. Compared with the case without *V*_*pc*_, the best latency is reduced by 19% for *t*_99.0%_ (@*V*_*pc*_ of 0.3), 13% for *t*_99.5%_ (@*V*_*pc*_ of 0.2), and 13% for *t*_99.9%_ (@*V*_*pc*_ of 0.3). The convergence time is increased when *V*_*pc*_ above a certain value is applied. < *f*_*SNN*_ > *_norm_* in the output layer for *Net 2* with respect to the different levels of *PCMP* from 0.0 to 0.5 is illustrated in Figure 5C. The early rising of < *f*_*SNN*_ > *_norm_* is observed as *V*_*pc*_ increases; however, for the case with *V*_*pc*_ above 0.3, there occurs a fluctuation by over- and under-firing caused by too much *PCMP*, which is the cause of the increase in the latency.

**FIGURE 6:**
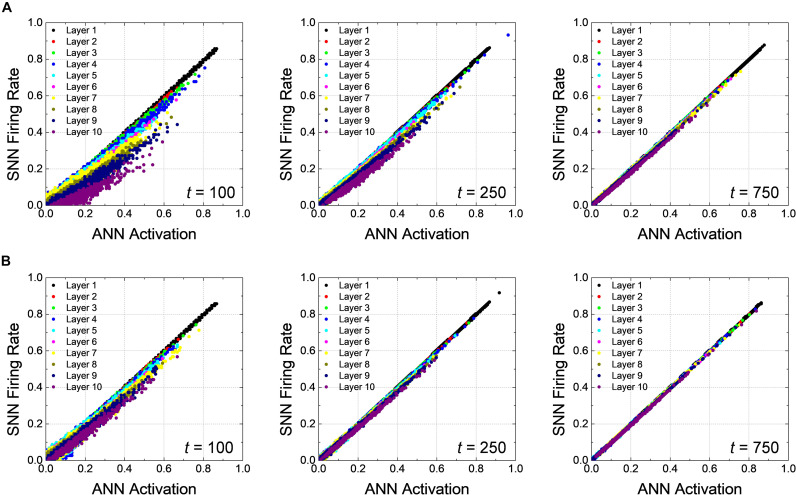
Correlation diagrams of *Net 2* at 100, 250, and 750th timesteps for the global *V*_*pc*_ of **(A)** 0.0 and **(B)** 0.3. In a deep network, the latency is more clearly seen compared with the case of *Net 1*.

The correlation diagrams of *Net 2* show the effect of the latency reduction more clearly than that for *Net 1*. Figures 6A,B represent the correlation diagrams of all neurons at the 100, 250, and 750th timesteps using all test data with *V*_*pc*_ of 0.0 and 0.3, respectively. In deep SNNs, as expected, the latency appears more pronounced for the neurons closer to the output layer, as illustrated in Figure 6A. The firing rates gradually increases so that it approaches the ANN activations, but it takes more time compared with the case of *Net 1*. The slope quickly reaches to one for *V*_*pc*_ of 0.3 compared with the case for *V*_*pc*_ of 0.0. Eventually, all the points are well-correlated to the ANN’s activations for the case using *V*_*pc*_ of 0.3 at the 750th timestep, while the latency still remains in the case of *V*_*pc*_ of 0.0 at the same timestep.

**FIGURE 7:**
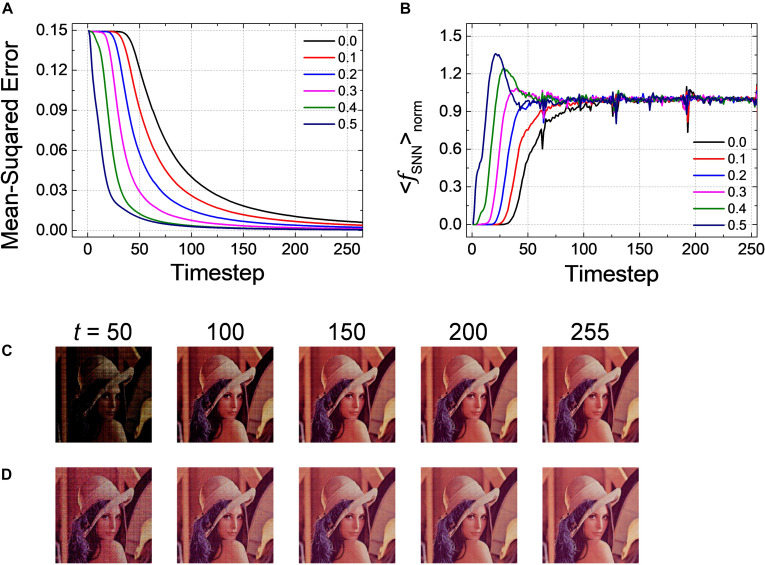
**(A)** Changes of *MSE* for *Net 3* when increasing the amount of the global *V*_*pc*_ from 0.0 to 0.5 with 0.1 steps. *MSE* decreases more quickly as the global *V*_*pc*_ increases. **(B)** The average firing rate in the output layer with the different levels of the global *V*_*pc*_ according to the simulation timestep. Restored sample image in the output layer of *Net 3* at 50, 100, 150, 200, and 255th timestep for the global *V*_*pc*_ of **(C)** 0.0 and **(D)** 0.5.

*Pre-charged membrane potential* is effective to reduce the latency as well not only for a classification problem but also for an autoencoder as *Net 3*. In particular, unlike the classification problems in which the relative number of output spikes matters, the absolute firing rate itself is of importance in autoencoder. Figure 7A shows the changes of *MSE* as a function of the inference time in the output layer with *V*_*pc*_ varying from 0.0 to 0.5. *MSE* gradually decreases as a function of the inference time, but it converges much faster when the amount of *PCMP* increases. The early firing of spikes and fast convergence are also observed by < *f*_*SNN*_ > *_norm_* with respect to the inference time for the output layer with the different levels of *PCMP*, as shown in Figure 7B. The changes of a decompressed image in the output layer at the 50, 100, 150, 200, and 255th timesteps for *V*_*pc*_ of 0.0 and 0.5 are shown in Figures 7C,D, respectively. In both cases, the images at the 255th timestep are successfully restored close to the original image; however, compared with the images using *V*_*pc*_ of 0.0, the images using *V*_*pc*_ of 0.5 become similar to the original image more quickly.

### Optimization of Pre-charged Membrane Potential

The global *PCMP* that applies the same amount of *V*_*pc*_ to all neurons in the network is a practical approach because of its simplicity, but we may optimize the amount of *PCMP* layer by layer. Brute-force layer-wise optimization, however, requires extraordinary computational power. For example, if there are 10 layers and 10 possible *V*_*pc*_ values for each layer, we have to check 10^10^ cases, each of which would take at least 10 s with the help of a powerful graphical processing unit (GPU). The total amount of time we have to devote for this task would be more than 300 years.

Based on the observation that the SNN error, $E_{i}^{l}\,\left(t,V_{i}^{l}\,\left(0\right)\right)$, depends on the partial errors, $\varepsilon_{i}^{l}$, for *l* ∈ {0,…,*L*}, we have devised a sequential layer-wise optimization method. Here, we assume that all the neurons in layer *l* have the same initial membrane potential, so $V_{i}^{l}\,\left(0\right)$ can be denoted as *V^l^*. For layer-wise optimization, we define the mean-squared SNN error ⟨[*E^l^*(*t*,*V^l^*)]2⟩ in layer *l* for the training set, which is expressed as

(6) $$\left\langle {\left[E^{l}\,\left(t,V^{l}\right)\right]}^{2}\right\rangle =\frac{\sum_{i=1}^{M^{l}}{\left[E_{i}^{l}\,\left(t,V^{l}\right)\right]}^{2}}{M^{l}}$$

As a first step, ⟨[*E*^0^(*t*,*V*^0^)]^2^⟩ is extracted by changing the initial membrane potential *V*^0^ from 0.0 to 0.5 with 0.01 steps. Then, *V*^0^ which minimizes the time sum of ⟨[*E*^0^(*t*,*V*^0^)]^2^⟩ during 1-epoch is determined as the optimal $V_{\text{pc}}^{0}$. Here, 255^*th*^ timestep is defined as one-epoch because the precision of the input image is eight-bit, so the input image is completely applied to the network at 255th timestep. This procedure can be repeated sequentially from the input to output layers. The point is that the optimization in layer *l* must be performed with the all the optimal *PCMP* in the previous layers ($V_{\text{pc}}^{0},V_{\text{pc}}^{1},\cdots ,V_{\text{pc}}^{l-1}$) applied. The layer-wise optimization procedure is described in Algorithm 1.

**Algorithm 1: Layer-Wise Optimization:** 

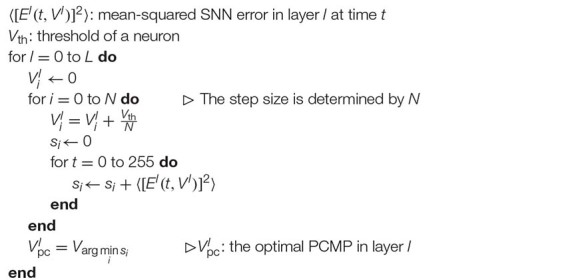

**FIGURE 8:**
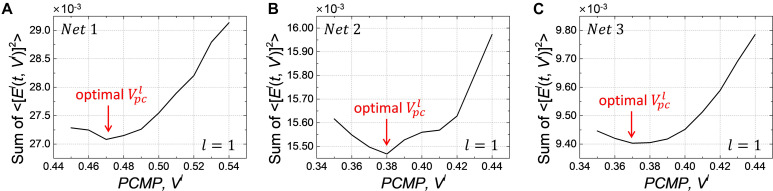
Changes of the time sum of ⟨[*E^l^*(*t*,*V^l^*)]^2^⟩ in layer *l=1* when increasing the amount of *PCMP*, *V^l^*for **(A)** *Net 1*, (**B)** *Net 2*, and **(C)***Net 3*. The optimization process is conducted from the input to the output layer sequentially, and it also needs to keep the optimized *PCMP*applied to the previous layers when performing the optimization for the subsequent layers.

**FIGURE 9:**
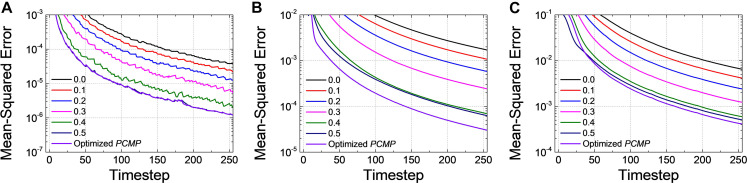
Changes of *MSE* for **(A)** *Net 1*, **(B)** *Net 2*, and **(C)** *Net 3* when increasing the amount of the global *V*_*pc*_ from 0.0 to 0.5 with 0.1 steps and the optimized *PCMP*. Although the global *PCMP* is practical due to its simplicity, the layer-wise optimization is more effective in a fast decrease of *MSE*.

We conduct the layer-wise optimization, and the time sum of ⟨[*E^l^*(*t*,*V^l^*)]^2^⟩ for *Net 1* ∼ *3* at *l=1*, as an example, is extracted according to *PCMP* in Figures 8A–C, respectively. It can be seen that the time sum of ⟨[*E^l^*(*t*,*V^l^*)]^2^⟩ decreases and then increases, so there is the global minimum of *V^l^* which is determined as the optimal $V_{\text{pc}}^{l}$. Figures 9A–C describe the changes of *MSE* between the SNN firing rates and ANN activations in the output layer of *Net 1* ∼ 3 over the simulation time by applying the global *PCMP* and the layer-wise optimization, respectively. It is confirmed that the layer-wise optimization results in a fast decrease of *MSE* compared with the case of the global *PCMP*. The latency of *Net 1* and *2* is summarized in Table 2, and the best record is marked in bold with ^∗^. In classification problems, it is found that a fast decrease in *MSE* does not necessarily guarantee rapid convergence in the accuracy since the accuracy is determined by comparing the relative number of spikes among the output neurons.

**TABLE 2:** Latency of Global *PCMP* and Layer-Wise Optimization for *Net 1, Net 2, and ResNet-20*.

**Network**	**Amount of *PCMP***		***t*_99.0%_**	***t*_99.5%_**	***t*_99.9%_**
*Net 1*	Global *PCMP*	0.0	17	19	32
		0.1	15	18	29
		0.2	12	15	27
		0.3	11	13	24
		0.4	9	12	29
		0.5	7	11	27
	Layer-Wise Optimization		6*	10*	19*
*Net 2*	Global *PCMP*	0.0	161	216	337
		0.1	139	207	311
		0.2	135	188*	296*
		0.3	132	205	460
		0.4	150	245	516
		0.5	176	267	762
	Layer-Wise Optimization		131*	204	513
*ResNet-20*	Global *PCMP*	0.0	408	511	703
		0.1	346	416	625
		0.2	261	343	497
		0.3	204	236	367
		0.4	154	194	239
		0.5	292	380	800
	Layer-Wise Optimization		152*	191*	238*

The best records in each network are marked in bold with *.

### Delayed Evaluation

There is a great fluctuation in the SNN error, $E_{i}^{l}\,\left(t,V_{i}^{l}\,\left(0\right)\right)$, during the initial timesteps. This is because the firing rate of input spikes has not reached the steady-state value. That is, a transient appears at the initial timesteps for SNNs. Spikes can be under-fired or over-fired during the initial transient, which contributes to the spike rate as significant errors. Therefore, if the inference operation is intentionally delayed in the output layer to eliminate the errors in the spike rate during the initial transient, fast and accurate inference operation can be achieved. We call this inference method as a *DE*.

**FIGURE 10:**
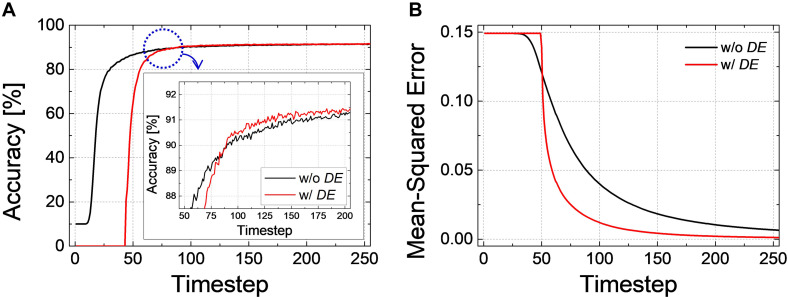
**(A)** Accuracy-timestep curve of *Net 2* with- and without *DE*. Both cases converge to the ANN’s performance, but the accuracy with *DE* converges faster than that without *DE*. **(B)** Changes of *MSE*with respect to the simulation timestep for *Net 3* with- and without *DE*. *MSE* with *DE* decreases and approaches zero much faster than that without *DE*.

As an example, the classification accuracy according to the simulation time for *Net 2* with *DE* of 40-timestep and without *DE* is illustrated in Figure 10A. During the intentional delay, the inference is not performed, so the classification accuracy cannot be extracted; however, it rapidly increases and converges to the ANN’s accuracy after the intentional delay. It may appear that the case with *DE* converges slower than that without *DE*, but if we closely examine the accuracy curves near the point where they cross each other, the accuracy with *DE* rapidly surpasses that without *DE*, as shown in the inset of Figure 10A. The effect of *DE* is also verified in *Net 3*. Figure 10B illustrates the changes of *MSE* with DE of 50-timestep and without DE. *MSE* remains constant during the early timesteps. Once the evaluation starts, however, *MSE* with *DE* decreases and approaches zero much faster than that without *DE*.

**FIGURE 11:**
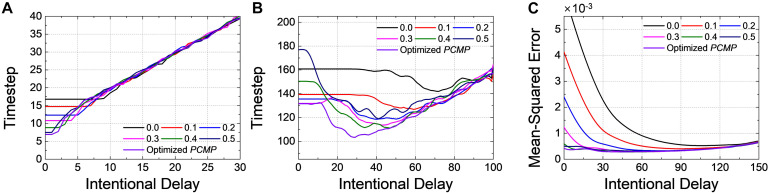
Changes of timestep when 99.0% of the ANN’s accuracy is reached with respect to the intentional delay for **(A)** *Net 1* and **(B)** *Net 2*. Changes of *MSE* at 1-epoch (255th timestep) for **(C)** *Net 3*. Different levels of the global and the optimized *PCMP* are used for *Net 1 ∼ 3*. Delayed evaluation (*DE*) is effective to achieve the further reduction of the latency, and it can be applicable simultaneously with *PCMP*.

We have investigated the inference performance by varying the intentional delay of *DE*. For example, Figure 11A shows the changes of timestep to reach 99.0% of the ANN’s accuracy (*t*_99.0%_) for *Net 1* with global and optimized *PCMP.* Firstly, *t*_99.0%_ shows no change when the intentional delay is short. In classification problems, the output neuron firing the most is considered as the correct answer; there is a period of time when no spike is generated in the output layer, so an intentional delay shorter than this “no-spike” period will not change the most-firing neuron; therefore, no change of accuracy is observed. If the intentional delay increases, however, *t*_99.0%_ increases as if it is pushed up by the excessive intentional delay. Even if the SNN is very close to the steady state, the evaluation time should be sufficiently long to achieve high accuracy; otherwise, the number of spikes corresponding to a low ANN activation would be too small for accurate evaluation. We can call this period of time “excessive intentional delay” period. The latency reduction by *DE* does not appear since *Net 1* has a short latency due to the relatively simple network structure and data to classify.

For *Net 2* with global and optimized *PCMP*, *t*_99.0%_ is extracted with respect to the intentional delay, as shown in Figure 11B. As mentioned above in Figure 11A, there occurs “no-spike” period as well in *Net 2*. After the no-spike period, spikes start to be generated in the output layer, but the spike rate is still incorrect. By *DE*, we can remove errors in the spike rate, which helps to decrease *t*_99,0%_. Thus, this period of time where *t*_99.0%_ decreases is referred as “error removal” period. Here, we can notice that that the combined use of *PCMP* and *DE* is significantly effective in reducing the latency. With large the intentional delay, however, *t*_99.0%_ suffers from “excessive intentional delay” period, it is dominated by the intentional delay.

For Net 3 with global and optimized *PCMP* and *MSE* at 1-epoch (255th timestep) is extracted by changing the intentional delay, as illustrated in Figure 11C. Unlike the classification problem, *MSE* decreases rapidly with the increase of *DE*. Since *DE* excludes a chunk of “no-spike” period in evaluation, the output spike rates get closer to the target spike rates rapidly. As *DE* increases further, however, *MSE* remains more or less constant. This is because the precision reduced by *DE* induces errors, but there is also the error reduction due to the removal of the initial transient. Those effects cancel each other out, resulting in little changes of *MSE*. After that, *MSE* increases since it starts to be in “excessive intentional delay” period.

### Effects on *ResNet-20*

In order to demonstrate the effectiveness of the proposed methods in much deeper and most generally used neural network models, we train *ResNet-20* using CIFAR-10 dataset (He et al., 2016). Firstly, the network is trained by SGD algorithm with the momentum of 0.9 and L2 weight decaying parameter of 1 × 10^–4^. Adaptive learning rate is employed with the initial learning rate of 0.1, so it is multiplied by a factor of 0.1 after 82, 123, and 164 epochs. Also, 32 × 32 random cropping (padding = 4) and the horizontal flipping are used for data augmentation. We can obtain the accuracy of 91.82% for the test dataset. The trained network is converted to SNN using the weight normalization method of *ResNet* which has been already reported in the previous research (Hu et al., 2018).

**FIGURE 12:**
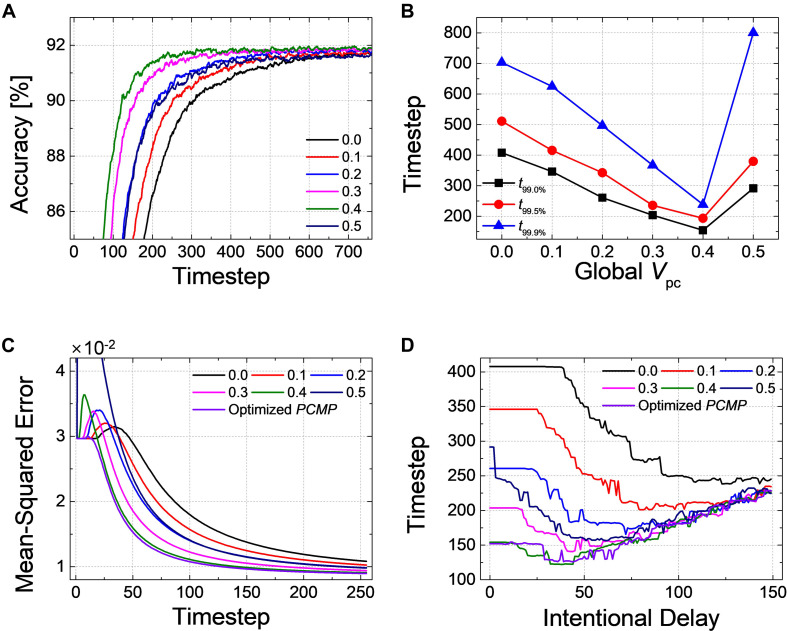
Effects of the proposed methods on *ResNet-20*. **(A)** Changes of the classification accuracy according to the global *V*_*pc*_ from 0.0 to 0.5. **(B)** Extracted timesteps (*t*_99.0%_, *t*_99.0%_, and *t*_99.9%_) with respect to the amount of the global *V*_*pc*_. **(C)** Changes of *MSE* for when increasing the amount of the global *V*_*pc*_ from 0.0 to 0.5 with 0.1 steps and the optimized *PCMP*. **(D)** Changes of timestep when 99.0% of the ANN’s accuracy is reached when using *DE*.

Figure 12A shows the accuracy of the spiking *ResNet-20* according to the simulation timestep with the different value of the global *PCMP*. When increasing the global *PCMP* from 0.0 to 0.4, the curve shifts to the left, and it goes back to the right when the global *PCMP* of 0.5 is applied. We extract *t*_99.0_, *t*_99.5_, and *t*_99.9%_ with the different amount of the global *PCMP*, as illustrated in Figure 12B. For all criteria, it is found that the convergence time decreases with the increase of the global *PCMP* up to 0.4 and increases again at the global *PCMP* of 0.5. Figure 12C indicates the result of *MSE* in the output layer when the layer-wise optimization is performed. *MSE* with the optimized *PCMP* appears smaller than that with the global *PCMP*. The best records of the latency in *ResNet-20* with *PCMP* are summarized in Table 2. We also perform the DE in *ResNet-20*, and *t*_99.0%_ is extracted with respect to the intentional delay, as shown in Figure 12D. It is confirmed that the convergence time remains the same during “no-spike” period, decreases during “error removal” period, and increases during “excessive intentional delay” period. These results are consistent with the results above; therefore, the effectiveness of the proposed methods is successfully demonstrated in much deeper networks.

## Discussion

### Reduction of Computational Cost

**FIGURE 13:**
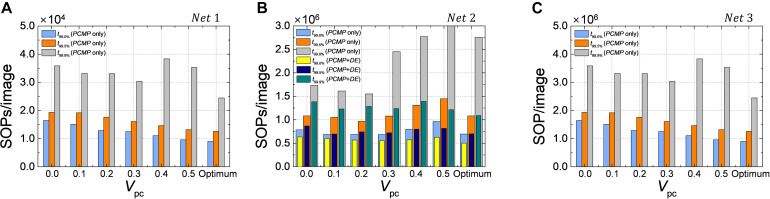
The number of synaptic operations per image (SOPs/image) for **(A)** *Net 1*, **(B)** *Net 2*, and **(C)** *Net 3*. SOPs/image is extracted based on *t*_99.0%_, *t*_99.0%_, and *t*_99.9%_ in the classification problems and on the reference *MSE* (*MSE*@1-epoch without *PCMP* and *DE*). The effect of the proposed methods for energy-efficient computing is remarkable.

The one of the most powerful advantage in using SNN is that energy-efficient inference is possible due to its event-driven characteristic. SNN updates its state when there is spike; therefore, the computational cost is proportional to the number of synaptic operations, which can be approximated by the number of spikes when performing the inference. Reducing the latency of SNN can contribute to saving of the number of synaptic operations required to determine the result. In order to confirm the advantage of the proposed methods in terms of the computation cost, we extract the number of synaptic operations per image (SOPs/image) for *Net 1* ∼ *3*, as shown in Figure 13. For classification problems such as *Net 1* and *2*, SOPs/image is extracted based on the latency to reach 99.0, 99.5, and 99.9% of the ANN’s accuracy (*t*_99.0_, *t*_99.5_, and *t*_99.9%_). In the case of autoencoder, such as *Net 3*, *MSE* at 1-epoch is set as a reference when neither *PCMP* nor *DE* is applied, and SOPs/image is extracted based on the latency to reach the reference *MSE*.

Because *DE* has no effect on the latency reduction in *Net 1*, Figure 13A shows SOPs/image for *Net 1* only using the global *PCMP* (0.0 ∼ 0.5) and optimized *PCMP* by the layer-wise optimization. It is clearly confirmed that the latency reduction by *PCMP* leads to the reduction of SOPs/image. The best computational cost is obtained when the layer-wise optimization of *PCMP* is performed, where it is reduced by 46, 35, and 32% for *t*_99.0_, *t*_99.5_, and *t*_99.9%_, respectively, compared with the case without *PCMP* and *DE*. For *Net 2*, as shown in Figure 13B, when only *PCMP* is applied, the best SOPs/image is obtained at *V*_*pc*_ of 0.2 for *t*_99.9_ and *t*_99.5%_ and at the optimized *V*_*pc*_ for *t*_99.0%_, which are reduced by 11, 11, and 12%, respectively, compared with the case without *PCMP*. Moreover, it is obviously demonstrated that the combination of *PCMP* and *DE* has a significant effect on reducing the computation cost so that it helps to reduce SOPs/image by 37, 36, and 38% for *t*_99.0_, *t*_99.5_, and *t*_99.9%_, respectively, compared with the case without *PCMP* and *DE.* The effect of *PCMP* and *DE* on the reduction of the computational cost is verified as well in the autoencoder, *Net 3*, the best SOPs/image to reach the reference *MSE* is reduced by 74% when the optimized *V*_*pc*_ and *DE* are applied, as shown in Figure 13C.

### Comparison With Prior Work

Table 3 shows the comparison results with the prior work in terms of dataset, spike encoding, accuracy, architecture, accuracy, and the latency. In the previous research implementing CNN through rate-based coding, the latency of 200-timestep was required to achieve the accuracy of 99.10% for MNIST (Diehl et al., 2015). However, it is possible to reach the accuracy of 99.25% for MNIST with the latency of 19-timestep based on the same spike encoding method by using the proposed methods. There also have been various studies implementing SNNs for MNIST pattern recognition using a spike encoding method other than rate-based coding (Kim et al., 2018; Park et al., 2019; Park et al., 2020). Most of the studies showed sufficiently high accuracy (above 99.00%), and, especially, the spike encoding method called the weighted spike showed the best performance with the latency of 8-timestep (Kim et al., 2018). The network for CIFAR-10 classification has a more complex network than that for MNIST, so it generally shows a longer latency. *Net 2* in this work shows better accuracy compared to the comparison groups, and its latency is also generally the same or better than other encoding methods. In addition, the proposed methods for *ResNet-20* is as effective as the previously reported method using FS-coding on the same structure resulting in the comparable latency (Stöckl and Maass, 2020). Among the previous studies for CIFAR-10, the weighted spike scheme showed the shortest latency of 42-timestep. In the case of autoencoder, the latency is extracted based on the timestep to reach the reference *MSE* as mentioned above. However, since most of the SNN-related studies focus on implementing the classification problem, there are not enough studies to compare. Comparing several aspects of the performance with the prior studies may be somewhat unfair because not all the networks have the same architecture. Even so, the proposed methods in this work are applicable to any architecture of SNN, and they still have an important meaning for implementing low-latency SNN because they show comparable or better performance compared with other methods in the previous studies.

**TABLE 3:** Comparison with prior work.

**Dataset**	**Spike encoding**	**Architecture**	**Accuracy [%]**	**Latency [timestep]**
*MNIST*	Rate (Diehl et al., 2015)	12**C**5-**P**2-64**C**5-**P**2-**FC**10	99.10	200
	Weighted spike (Kim et al., 2018)	12**C**5-**P**2-64**C**5-**P**2-**FC**10	99.20	8
	Time-to-first-spike (Park et al., 2020)		99.33	40
	Burst (Park et al., 2019)		99.25	87
	**Rate (our method, *PCMP)***	***Net 1***	**99.25**	**19**
*CIFAR-10*	Rate (Rueckauer et al., 2017)	32**C**3-32**C**3-**P**2-64**C**3-64**C**3-**P**2-**FC**512-**FC**10	87.82	280
	Weighted spike (Kim et al., 2018)	32**C**3-32**C**3-**P**2-64**C**3-64**C**3-**P**2-**FC**512-**FC**10	89.10	42
	FS-coding (Stöckl and Maass, 2020)	*ResNet-20*	91.45	200
	Time-to-first-spike (Park et al., 2020)	VGGNet-16	91.43	680
	Burst (Park et al., 2019)	VGGNet-16	91.41	793
	**Rate (our method, *PCMP)***	***Net 2***	**91.67**	**296**
	**Rate (our method, *PCMP* + *DE)***	***Net 2***	**91.67**	**204**
	**Rate (our method, *PCMP)***	***ResNet-20***	**91.72**	**238**
	**Rate (our method, *PCMP* + *DE)***	***ResNet-20***	**91.72**	**198**
*CIFAR-10 (Autoencoder)*	**Rate (our method, *PCMP)***	***Net 3***		**58**
	**Rate (our method, *PCMP* + *DE)***	***Net 3***		**56**

*Bold values indicates the best records by our methods.*

## Conclusion

In this work, methods to reduce the inherent latency in SNN are proposed, which is applicable at the inference stage. The membrane potential of a neuron is charged to some extent prior to inference operation, which is denoted as the pre-charged membrane potential (*PCMP*). Also, we introduce a deliberate delay in the output layer to discard some spikes occurring at the initial timesteps, referred as a *DE*. It is demonstrated through the model equations of SNNs that *PCMP* reduces the SNN error by inducing the earlier firing of the first spike, and *DE* eliminates the error spikes at the early timesteps so that low-latency SNN can be achieved. In SNN applications, such as classification and autoencoder, the proposed methods successfully reduce the latency, the combined use of *PCMP* and *DE* can help to achieve further latency reduction. Moreover, required the synaptic operations are significantly improved by using the proposed methods, which leads to energy-efficient computing.

## Data Availability Statement

The original contributions presented in the study are included in the article/Supplementary Material, further inquiries can be directed to the corresponding author/s.

## Author Contributions

SH, JC, M-HO, and KM: conceptualization. TJ, KP, and JY: data curation. SH: writing—original draft preparation. SH and B-GP: writing and editing. J-HL and B-GP: supervision. B-GP: project administration. All authors have read and agreed to the submitted version of the manuscript.

## Conflict of Interest

The authors declare that the research was conducted in the absence of any commercial or financial relationships that could be construed as a potential conflict of interest.
